# Human Species D Adenoviruses Isolated from Diarrheal Feces Show Low Infection Rates in Primary Nasal Epithelial Cells

**DOI:** 10.3390/children8070563

**Published:** 2021-06-30

**Authors:** Malik Aydin, Sebastian Schellhorn, Stefan Wirth, Wenli Zhang, Anja Ehrhardt

**Affiliations:** 1 Laboratory of Experimental Pediatric Pneumology and Allergology, Center for Biomedical Education and Research, School of Life Sciences (ZBAF), Faculty of Health, Witten/Herdecke University, 58455 Witten, Germany; 2 Center for Child and Adolescent Medicine, Helios University Hospital Wuppertal, Witten/Herdecke University, 42283 Wuppertal, Germany; stefan.wirth@uni-wh.de; 3 Virology and Microbiology, Center for Biomedical Education and Research (ZBAF), Department of Human Medicine, Faculty of Health, Witten/Herdecke University, 58455 Witten, Germany; sebastian.schellhorn@uni-wh.de (S.S.); wenli.zhang@uni-wh.de (W.Z.); anja.ehrhardt@uni-wh.de (A.E.)

**Keywords:** adenovirus, virus, infection, nasal epithelial cells, transduction, CAR, CD46

## Abstract

The importance of adenovirus (Ad) research is significantly increasing with respect to virotherapy for vaccine development, tumor, and gene therapy. Due to the different species and subtypes of this virus, the characterization of the biological significance of especially rare Ad is necessary. Previously, rare Ad types 70, 73, and 74 were originally isolated from fecal samples of immunocompromised patients and they represent recombinants of other Ad types. Here we investigated transduction experiments of these reporter gene tagged Ad types in primary cells exemplified by subject-derived primary nasal epithelial cells (NAEPCs). To analyze the transduction rates, we performed flow cytometry, quantitative polymerase chain reaction (PCR), and cytokine analyses 25 h post-infection. We found that, in contrast to Ad type 5 (as a positive control), the transduction rates of NAEPCs with Ad types 70, 73, and 74 were interestingly low. The major Ad receptor (coxsackievirus-adenovirus receptor and CD46) expression levels showed no significant change after infection with Ad types 70, 73 and 74. Moreover, Interleukin 6 (IL-6) was not released after in vitro Ad transduction. Due to the high risk of developing life-threatening complications in immunocompromised patients by these human species D Ads, even more attention needs to be investigated into the development of diagnostic and therapeutic concepts to prevent and treat those opportunistic infections in susceptible patients.

## 1. Introduction

Adenoviruses (Ads) are non-enveloped, icosahedral double-stranded DNA viruses, with genome sizes between 26 to 45 kilobase pairs (kbps) [1]. For host cell infection, they utilize different cell surface molecules, including the coxsackie and adenovirus receptor (CAR), cluster of differentiation (CD46), desmoglein-2, GD1a ganglioside and sialic acid residues for virus entry usually followed by integrin-mediated virus internalization, which explains the broad tissue tropism of Ads [2]. Currently, the known human Ads are subdivided into seven species (A–G) with different hemagglutination properties, oncogenicity, DNA homology and genomic organization [3,4]. Ads are used in gene therapy to treat genetic disorders such as cystic fibrosis, as vector for vaccination studies or in virotherapy to treat cancer [5,6,7,8]. However, Ads can also be pathogenic causing well-known clinical symptoms as for instance in the eye (keratoconjunctivitis), the gastrointestinal (gastroenteritis) and the respiratory tract [9,10,11,12,13,14]. Although these infections are usually self-limiting, Ads can also cause fatal disseminating diseases in immunocompromised patients [15,16,17,18,19,20]. Up to now, 104 distinct Ad types were identified (http://hadvwg.gmu.edu, accessed on 25 March 2021), and for many of these types limited information is available. Thus, research is needed to better understand the virus-host-interactions in vitro and in vivo. In 2015, Hage and colleagues characterized for the first time a new Ad type 70 from stool samples of a stem cell transplanted patient with diarrhea [21]. They determined its complete genome to consist of 35,186 base pairs (bps) coding for 39 putative open reading frames (ORFs). While only its fiber region was almost completely identical to another species D virus, Ad type 29, most other regions including the penton, hexon as well as the E3 and E4 gene regions showed significant differences to the counterparts in their most closely related human Ads of species D [21]. Two years later, additional Ad types 73, 74, and 75 were identified by the same group from the stool samples of patients with a lymphoma, with allogeneic stem cell transplantation for myelodysplastic syndrome, and from stool samples of an AIDS patient [22]. All four Ads were not found to be closely related to one another on the complete genome level, but on single gene and gene region level, e.g., penton, E1 and E4 [22].

In our previous study, we found that Ad5, among others, showed the best transduction efficiency in primary, human nasal epithelial cell (NAEPCs) cultures and that major Ad receptors such as CAR and CD46 were expressed on these cells. [23]. Considering this observation, we sought whether fecal Ads could pass through the respiratory tract with or without infecting the upper organs before entering the gastrointestinal system and causing life-threating infections or gastrointestinal symptoms in e.g., immunocompromised patients. Previously, we cloned Ad types 70, 73, and 74 from fecal samples and tagged them with reporter genes such as nano-luciferase and enhanced green fluorescent protein (GFP) [3]. In this previous study, we found that, in general, transduction efficiencies were comparably low in various immortalized cancer cell lines. Furthermore, we found that these viruses utilize the CD46 entry receptor and that Ad74 also uses CAR for attachment and entry [3]. The recombinant Ad types 70, 73, and 74 (Ad70GLN, Ad73GLN, and Ad74GLN), which contain a dual-reporter cassette (GLN) including GFP and luciferase inserted into E3 region in reverse direction to the Ad major late prompter (MLP), have the same phenotype as individual wild type virus. Therefore, they also cause cytopathic effect (CPE) in A549 cells. It is of note, the recombinant viruses were rescued in HEK 293 cells, but they all can also replicate efficiently in A549 cells [3].

Viruses, including Ads can be involved into the activation of pro-inflammatory processes by secreting e.g., cytokines and chemokines, where their release may lead to innate and adaptive immune responses [24,25,26]. An uncontrolled activation such as a ‘cytokine storm’ can cause serious and harmful processes for the host [24,25,26,27]. Interleukin-6 is an acute phase inflammation parameter and is secreted during infection, injuries or can be also involved in cancer development and proliferation [28,29]. Its release is measurable in the systemic blood and can provide important notes for sepsis [26,30,31].

To obtain a first approximation step on the passageway of these species D viruses, we aimed at analyzing the transduction efficiencies of these species D viruses in primary cells such as human nasal epithelial cells (NAEPCs) and investigated the viral effects on the major receptors CAR and CD46.

## 2. Results

### 2.1. Transduction Efficiencies of Ad 70, 73, and 74 Were Dose-Dependent in NAEPCs

Recently, three additional Ads including Ad 70, 73, and 74 have been isolated from fecal samples and successfully tagged with a green-fluorescent protein (GFP) and nano- luciferase [3]. First, we hypothesized whether the transduction efficiency in NAEPCs can be dose-dependent. For this, different multiplicity of infection (MOI) of 10, 100, and 1000 were used, and the cells were transduced for 25 h. As previously shown [23], the transduction efficiency of Ad5 in NAEPCs was superior. Therefore, this information was used as a ‘positive control’ to compare the following results with Ad 70, 73, and 74.

### 2.2. Adenoviruses 70, 73, and 74 Showed Low Transduction Efficiency in NAEPCs

**Figure 1:**
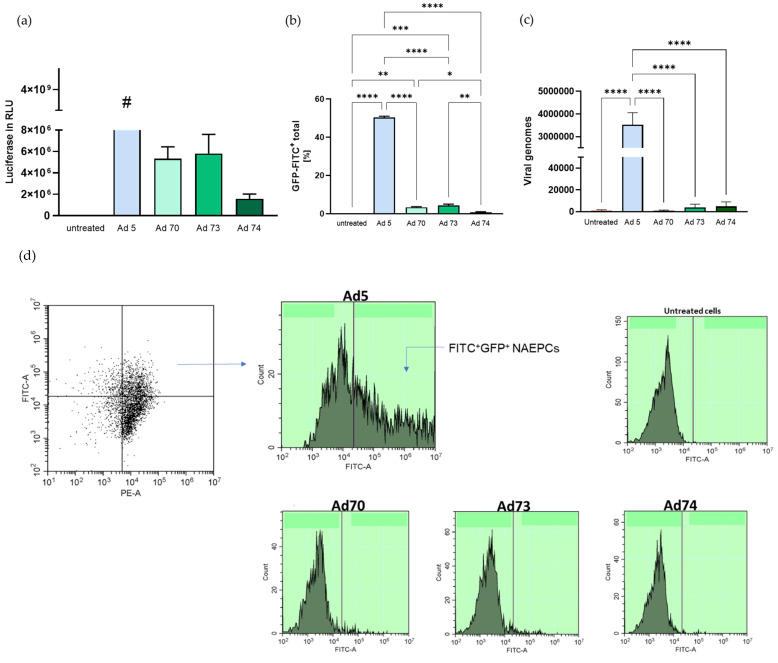
Transduction efficiencies of adenovirus (Ad) types 70, 73 and 74 in human nasal epithelial cells (NAEPCs) compared to Ad5. To study the transduction efficiency of Ad70, 73 and 74, NAEPCs obtained from healthy donors were infected at a multiplicity of infection (MOI) of 100. After 25 h, luciferase assays, flow cytometric analyses were performed, and DNA was extracted for quantitative PCR analyses. (**a**) By luciferase assay, the transduction efficiency of the Ad 5, 70, 73, and 74 was analyzed. This viral susceptibility of Ad 5 was set as ‘positive control’. Compared to Ad 70, 73, and 74, the relative light units (RLU) were reduced compared to Ad 5. **#** Due to unmeasurable values (over the measurable range in software program) of Ad 5, a statistical analysis was not performed and the results of Ad5 are fictively presented as a ‘maximum value’. (**b**,**d**) Flow cytometry was performed to measure the transduction efficiency; here green fluorescent protein positive (GFP^+^) NAEPCs were low for Ad 70, 73, and 74 as compared to Ad5. (**c**) Quantitative PCR of the virus genomes in NAEPCs was performed. Values are given as mean and standard error of the mean (*n* = 3 biological replicates, with each different technical replicates as stated in the Supplementary Section), (* *p* < 0.05, ** *p* < 0.01, *** *p* < 0.001, and **** *p* < 0.0001).

The viral susceptibility of the cells was analyzed by luciferase assay, flow cytometry, and quantitative PCR analyses. For this purpose, cells were transduced with Ad5, 70, 73, and 74 at an MOI of 100 for 25 h. By means of luciferase assay, non-measurable very high relative light units (RLU) for Ad5 were measured, whereas these values were comparatively low for Ad 70, 73, and 74 (Figure 1a). In addition, flow cytometry was applied to measure GFP positive cells. Compared to Ad5 and non-treated cells, less GFP^+^ cells were measured (Figure 1b,d). Furthermore, viral genomes in the NAEPCs were analyzed by quantitative PCR analysis. Here, the viral copy number for Ad70, 73 and 74 was significantly lower compared to Ad5 (Figure 1c).

### 2.3. Infection With Adenoviruses 70, 73, and 74 Does Not Significantly Influence Viral Entry Receptors

**Figure 2:**
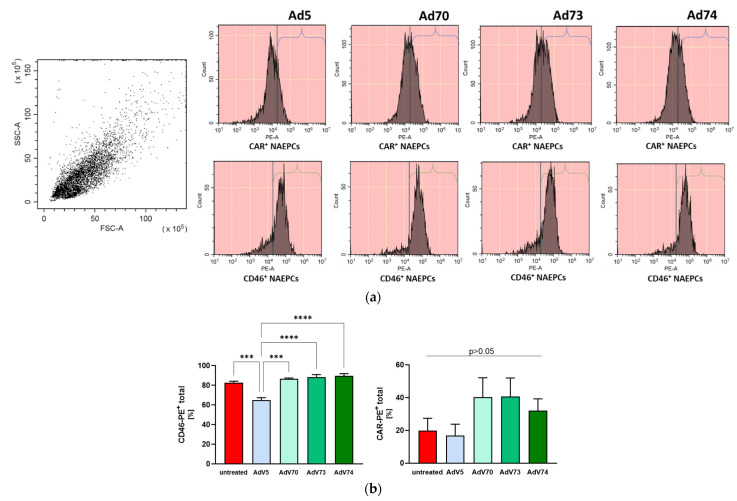
The expression of CAR and CD46 was not significantly influenced by Ad 70, 73 and 74. (**a**) Primary human nasal epithelial cells (NAEPCs) were transduced with Ads 5, 70, 73, and 74 at an MOI of 100 and characterized 25 h post-infection by flow cytometry for CAR and CD46 expression levels. (**b**) Transduction with Ad 70, 73 and 74 was not associated with any significant up- or downregulation of these receptors compared to Ad 5 or untreated cells. Values are given as mean and standard error of mean (*n* = 3 biological replicates), (*** *p* < 0.001, and **** *p* < 0.0001).

Adenoviruses use distinct receptors, including CD46 and CAR, to enter and infect cells [32]. Sakurai and colleagues showed a time- and dose-dependent downregulation of CD46 upon Ad 35 infection [33]. Thus, we addressed the question whether Ad 70, 73, and 74 may influence the receptor expression levels of CD46 and CAR on NAEPCs. NAEPCs were transduced with Ad 5, 70, 73 and 74 at an MOI of 100, and were characterized by flow cytometry 25 h post-infection. Neither CAR nor CD46 showed any significant over- or downregulation of these receptors after transduction with Ad 70, 73 and 74 compared to uninfected control. However, for Ad5 we observed a significant downregulation of CD46 if directly compared to the other analyzed groups (Figure 2a,b).

### 2.4. Interleukin-6 Is Not Significantly Influenced by Adenoviruses 5, 70, 73, and 74

**Figure 3:**
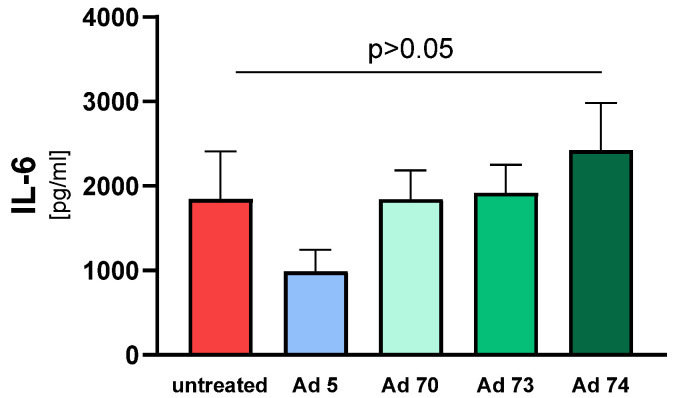
An in vitro transduction of Ads 5, 70, 73, and 74 does not lead to a significant change of IL-6 concentration levels. After cultivation of NAEPCs and 25 h-transduction with Ads 5, 70, 73, and 74, supernatants were used for IL-6 measurement through LEGENDplex^TM^ assay (Biolegend, San Diego, CA, USA). Values are given as mean and standard error of mean (*n* = 3 biological replicates with each *n* = 3 technical replicates).

To analyze whether the concentration of IL-6 is released or decreased upon infection with Ads, we analyzed IL-6 concentrations in supernatants of transduced samples. After 25 h in vitro transduction of NAEPCs, supernatants were used for further cytokine measurement. The transduction of Ad 5 presented a lower concentration level in these samples compared to other samples transduced with Ad 70, 73, and 74. Note that these results were statistically not significant (Figure 3).

## 3. Discussion

In this study, we investigated the transduction efficiency of Ad 70, 73, and 74 on NAEPCs, which have not yet been extensively studied in detail. In summary, we found that the transduction efficiency of these viruses in NAEPCs was low. Moreover, we did not observe any significant changes of CD46 and CAR after Ad transduction in vitro.

Several vaccination trials on Influenza were successfully performed using Ad5 as a vector in animals (e.g., [34,35]). However, due to the significant seroprevalence against Ad5, vaccination studies may fail in humans [35,36]. Interestingly, human species D Ads are successfully used for developing vaccines e.g., against SARS-CoV-2/Covid-19 [35]. Thus, the role of human species D Ads needs to be studied in more details in the nasal epithelium with the potential aim to develop nasal vaccines.

In our previous work, the transducibility of these Ads was investigated in different cell lines, including different breast cancer lines, HEK293- and HeLa cells, A549- lung cancer cells and colon cancer cells (HCT116 and Caco2 cells) [3]. In this study, it was observed that transduction efficiencies of Ad 70, 73, and 74 were low in A549-, HeLa-, and HEK293 cells, and compared to Ad5, also significantly reduced in colon, hepatocellular carcinoma- and breast cancer-derived cell lines [3]. However, less information is yet presented on the exact passageway of these Ads to the gastrointestinal tract. Therefore, assuming that these Ads enter the gastrointestinal tract through the naso-pharyngeal route, we intended to determine whether these viruses also infect NAEPCs. If this hypothesis were to be verified, another hypotheses would be which entry receptors these viruses primarily use and whether nasal vaccination trials can be potentially established to protect immunocompromised patients from serious complications. In detail, with this brief report, we aimed at investigating how these gastrointestinal rare Ads infect these cells during the passage from the upper organs (including respiratory tract). Considering this hypothesis, with this approach, we here used primary cells from the respiratory tract to analyze the effects of Ad 70, 73, and 74 on NAEPCs.

In the literature, Ads target different receptors to attach to and infect cells [37,38,39]. Several studies revealed that the respiratory epithelium expresses CD46 and CAR [40,41] and infection by respiratory Ads including Ad5 is well studied in respiratory cells [42,43,44,45,46,47]. Nevertheless, the question arises why Ad 70, 73, and 74 did not transduce NAEPCs, although species D adenovirus also primarily use CD46 and CAR as viral entry [3,37,48]. Interestingly, we observed a downregulation of the CD46 receptor after Ad5 infection in NAEPCs as compared to Ad70, 73, and 74. This phenomenon was also observed by Sakurai and colleagues. They observed that replication-deficient Ad35 vectors led to downregulation of CD46 [33]. In addition, Gustafsson et al. also observed a downregulation of CD46 by Ad 11p [49]. Given this observation, we hypothesized that Ad5 downregulates CD46 to prevent infection with other viruses. It was also shown that the CAR receptor is downregulated in differentiated primary muscle and primary neurons [50,51]. However, our previous data revealed that both receptors are expressed on NAEPCs [23].

Our results showed that there was no significant change in IL-6 concentration in the supernatant after 25 h of transduction time. Interestingly, Qi and colleagues analyzed airway samples of children who were infected with Ad7 and observed that the concentration level of IL-6 in the nasal washes was superior in children infected with Ad7 compared to the healthy group [24]. Moro et al. (2009) also observed increased cytokine and chemokine levels including IL-6 in the nasal wash of children infected with Ads [52]. Either the concentration of IL-6 in supernatants of our samples was too low to get it detectable with that assay which we used, or the time point, and the concentration of the applied Ads played a role for the secretion of IL-6 in vitro. Thus, to prove this hypotheses, further follow-up experiments will be performed to conclusive the influence of Ads transduction and their relationship of IL-6 in vitro.

Nevertheless, how Ads 70, 73, and 74 overcome the passage to enter the intestinal tract and whether the upper respiratory tract acts ‘sends’ the viral load further towards the gastrointestinal tract is insufficiently understood yet. Although other gastrointestinal viruses such as rota- and noroviruses enter the human body through the fecal/oral route, there are examples of viruses, which can infect the respiratory- and the gastrointestinal tract. One prominent example is SARS-CoV2 [53] and there is an interesting example for the porcine epidemic diarrhea virus (PEDV) in swine, which can disseminate from the nasal cavity [54]. Although Ads 70, 73, and 74 were isolated from immunocompromised patients, there is no current evidence for a direct infection with these Ads. Other routes of infection or superinfection with different species D Ad types must be taken into consideration. Moreover, there can be distinct hypotheses whether these Ad 70′s showed low transduction rates in NAEPCs. Due to receptor usage or in general, all three species D viruses are moderate in infection that is why they can stay/persist in the gastrointestinal tract without causing any significant symptoms. To confirm this, further studies including translational approaches will be needed to study the role of Ad 70, 73, and 74. The receptor usage is also important to study the targeting processes and developing Ad-based vector vaccination [35]. Interestingly, Persson and colleagues studied the role of hexon capsid proteins and explored, that human species D Ad hexon capsid whether human species D Ad hexon capsid proteins use CD46 to enter the host [35].

Further experiments including primary gastrointestinal cells will show the molecular influence of the Ad 70, 73 and 74 on the gastrointestinal epithelium.

In summary, Ads 70, 73, and 74 are among the rare pathogens whose pathogenesis has not been fully explored. This work represents a first approach to study the function of these viruses on primary mammalian cells.

## 4. Materials and Methods

### 4.1. Cultivation of Primary Human Nasal Epithelial Cells

A detailed cultivation protocol of NAEPCs was previously described [23,55]. Briefly, deep-frozen NAEPCs of *n* = 3 healthy subjects were systematically thawed for this approach, washed twice with phosphate buffered saline (PBS) solutions, calculated, and seeding in either collagen type I-coated T25 or T75 flasks. Cells were harvested and seeded for experiments either in 24- or 96-well plates using a cell number of 20,000 or 10,000 cells per well. The next day, medium was removed, and wells were filled with fresh cell culture medium (BEGM^®^ Lonza, Basel, Switzerland) for transduction experiments. After approximately 4 h, the wells were replenished with cell culture medium (either to 200 µL in 96-well plates or 600 µL medium in 24-well plates). Luciferase assays were performed using cells seeded in collagen type I-coated 96-well plates. For flow-cytometricanalyses and for DNA extraction, cells were seeded in 24-well plates.

### 4.2. Adenoviruses Used in This Study

Reporter gene tagged recombinant adenoviruses used in this study were published previously [3]. In brief, a nano-luciferase and eGFP encoding transgene was inserted into the E3 region of the wild type Ad genome of Ad 70, 73, and 74 using an improved DNA recombineering technique [3,56]. Viruses were amplified in HEK293 cells and purified using cesium chloride gradients.

### 4.3. Luciferase Assay

Primary, human nasal epithelial cells were transduced with Ads 5, 70, 73 and 74 at different MOI rates. Twenty-five hrs. post-transduction, reporter gene expression was measured by luciferase assay using Nano-Glo^®^ Luciferase Assay (Lot: 0000449802) from Promega, Mannheim, Germany. For this, 150 µL medium was removed, 50 µL medium was remained which was mixed with 50 µL luciferase assay buffer and enzyme (1:50 diluted) and incubated for 5 min at 37 °C und 5% CO_2_. Then, the content of each well was transferred to a 96 well white plate with a solid bottom and measured using a plate reader (Tecan, Crailsheim, Germany) and the software i-control 2.0, INFINITE F-Plex. Values are given as relative light units (RLU) in mean and standard error of mean.

### 4.4. Determination of Viral Genomes though Quantitative Real-Time PCR

To analyze the viral genome numbers within the transduced NAEPCs, NAEPCs were transduced with Ad 5, 70, 73 and 74 at different MOI and incubated for 25 h. The next day, the medium was removed, and the cells were de-attached using trypsin and harvested. Then, cells were centrifuged (800× *g* for 6 min.), the supernatant was removed, and the cell pellet was stored at −80 °C. For DNA isolation, the DNA, RNA, and protein purification kit NucleoSpin^®^ purchased from MACHEREY-Nagel was used and the DNA isolation was performed according to the manufacturers’ instructions. The DNA was diluted in distilled water. The quantification of the viral genomes within the NAEPCs were performed through quantitative real-time PCR (CFX Connect Real-Time Detection System, BioRad, Germany) using forward/reverse primer as previously described [3,57] and my-Budget 5× EvaGreen^®^ QPCR-Mix II reagent (Bio-Budget, Krefeld, Germany) according to the manufacturer’s’ instructions.

### 4.5. Flow Cytometric Characterization of Viral Transduction Efficiency and Surface Receptors

Transduced NAEPCs were washed twice with PBS and treated with trypsin/EDTA. The reaction was stopped with cell culture medium and 10% fetal bovine serum. Cells were transferred into 1.5 mL sample tubes and centrifuged at 400× *g* for 6 min. The supernatant was decanted and resuspended in 100 µL PBS and 2% fetal bovine serum (FBS). The antibodies Anti-Hu-CD46 (PE, Clone BE2, Lot-NR: 1934008) were purchased from Invitrogen, eBiosciences and CXADR/CAR (10799-R271-P, HR13MY2802) from Sino Biologicals Inc. The antibodies were pipetted to the NAEPCs. After an incubation of 25 min, cells were centrifuged at 400× *g* for 6 min prior adding 1 mL PBS + 2% FBS. The supernatant was decanted, and the cell pellet was resuspended in 150 µL PBS/2%FBS. Flowcytometric characterization was performed using Cytoflex Beckmann Coultier. The FITC channel was assessed for GFP^+^ NAEPCs and the PE channel for CD46^+^ or CAR^+^ NAEPCs. Analyses were done using the analysis software of Cytoflex Beckmann Coultier.

### 4.6. LEGENDplex^TM^ Assay for IL-6 Measurement

Supernatants of transduced NAEPCs were used for IL-6 measurement. For this, LEGENDplex^TM^ assay by BioLegend, Canada was used, and the samples were prepared according to manufracturer’s instructions and as also described previously (https://doi.org/10.3389/falgy.2021.667562, accessed on 25 March 2021).

### 4.7. Statistical Analyses

For statistical analyses, GraphPad Prism version 9.0.2 for Windows, GraphPad Software, La Jolla, CA, USA, www.graphpad.com (accessed on 25 March 2021) was used. Values were given as mean or standard error of the mean. For statistical comparisons/differences between more than two groups, One-Way-ANOVA and Tukey’s multiple post-tests were used. The significance level was set at *p* < 0.05.

## 5. Conclusions

Adenoviruses can cause life-threatening complications in patients with e.g., chronic disorders. Thus, the role of novel and rare Ads needs to be further elucidated. The characterization of new Ads is associated with a great challenge, as their clinical and scientific significance must be worked out expeditiously in order to help affected subjects rapidly. With the description of these rare Ads, their utility in terms of gene therapy and vaccination is of particular interest. With this pilot work, we aimed to elaborate the infectivity of Ads 70, 73 and 74 in NAEPCs and aimed to obtain a first approximation about the passageway of these viruses. Further in vitro work should reveal how the passage pathway into the gastrointestinal tract occurs.

## Data Availability

The data to this study can be shared upon reasonable request from the corresponding author.

## Supplementary Materials

The following are available online at https://www.mdpi.com/article/10.3390/children8070563/s1, Tabular results of raw data of each subfigure.
