# Supplementary material for: Human Species D Adenoviruses Isolated from Diarrheal Feces Show Low Infection Rates in Primary Nasal Epithelial Cells
Source: Children (Basel). 2021 Jun 30;8(7):563. doi: 10.3390/children8070563 (PMC8307086; doi:10.3390/children8070563)
Supplement: Supplementary file 1 [file children-08-00563-s001.zip › children-1264636-supplementary.pdf]

Supplementary

Luciferase-Assay *(n = 3 biological replicates, incl. each n=9 technical replicates; for Fig. 1a)*

Untreated	Ad 5	Ad 70	Ad 73	Ad74
14751.5556	1e+009***	4183912	3852397.56	1216761.22
2074.55556	1e+009***	4296697.67	4198274.56	1054824
3658.33333	1e+009***	7529933.44	9373573.38	2474471

***values were over the measurable range as the software program can analyze it. Thus, these values of Ad 5 group are fictively listed (maximal values were chosen) and statistical analyses were not performed.

GFP-FITC^{+} total [%] *(n = 3 biological replicates, for Fig. 1b, d)*

untreated	Ad 5	Ad 70	Ad 73	Ad 74
0.09	51.24	3.2	4.18	0.85
0.07	50.7	3.94	5.49	1.05
0.06	49.05	2.9	3.28	0.49

Quantitative PCR viral genomes *(n = 3 biological replicates, incl. each n=3 technical replicates; for Fig. 1c)*

Untreated	Ad 5	Ad 70	Ad 73	Ad 74
2691.2494	4338589.26796	703.91319	9854.36791	13072.64975
3.57551	3713829.80178	2007.77735	1421.54901	972.00962
4.44935	2503066.24905	343.02894	361.61384	286.37386

CD46-PE [%] *(n = 3 biological replicates; for Fig. 2a, b)*

untreated	Ad 5	Ad 70	Ad 73	Ad 74
81.37	60.82	86.61	83.04	85.85
85.58	66.27	88.04	92.07	93.57
81.53	68.4	85.53	89.68	89.58

CAR-PE [%] *(n = 3 biological replicates; for Fig. 2a, b)*

untreated	Ad 5	Ad 70	Ad 73	Ad 74
21.93	14.92	47.06	53.01	40.48
31.78	29.8	56.57	50.87	37.95
5.97	5.98	17.36	18.03	17.96

IL-6 [pg/ml] *(n=3 biological replicates, incl. each n=3 technical replicates; for Fig. 3)*

**Untreated**	**Ad 5**	**Ad 70**	**Ad 73**	**Ad 74**
724.913333	526.506667	1201.95	1287.02333	1300.56667
2498.03	1417.17	2380.02	2050.55667	2951.07
2310.4	1023.09	1945.09333	2418.14	3014.16333
